# Overexpression of hypoxia-inducible-factor 1*α*(HIF-1*α*) in oesophageal squamous cell carcinoma correlates with lymph node metastasis and pathologic stage

**DOI:** 10.1038/sj.bjc.6601186

**Published:** 2003-09-09

**Authors:** T Kurokawa, M Miyamoto, K Kato, Y Cho, Y Kawarada, Y Hida, T Shinohara, T Itoh, S Okushiba, S Kondo, H Katoh

**Affiliations:** 1 Department of Surgical Oncology, Division of Cancer Medicine, Hokkaido University Graduate School of Medicine, Sapporo, Japan; 2 Department of Pathology, Teine Keijinkai Hospital, Sapporo, Japan; 3 Department of Surgical Pathology, Hokkaido University Hospital, Sapporo, Japan

**Keywords:** hypoxia-inducible-factor 1*α*, oesophageal squamous cell carcinoma, lymph node metastasis, immunohistochemistry, adjuvant therapy

## Abstract

The purpose of this study is to investigate the clinical and histopathologic significance of hypoxia-inducible-factor 1*α* (HIF-1*α*) expression in oesophageal squamous cell carcinoma. One hundred and thirty surgically resected specimens of OSCC were immunohistochemically assessed for HIF-1*α* expression with monoclonal antibody. High HIF-1*α* immunostaining was detected in 40 specimens. The percentage of high HIF-1*α* expression cases increased with tumour stage according to pTNM system. High HIF-1*α* expression correlated with pTNM stage, depth of tumour invasion, lymph node metastasis, distant metastasis, lymphatic invasion and positive surgical margin. The overall survival rate was worse in patients with high HIF-1*α* pattern than in patients with low-expression pattern. Univariate analyses identified high HIF-1*α* positivity, depth of tumour invasion, lymph node metastasis, distant metastasis, lymphatic invasion, and a positive surgical margin as risk factors. Multivariate analyses indicated that depth of tumour invasion, lymph node metastasis and positive surgical margin, but not HIF-1*α*, were independent prognostic factors. Survival in patients with a high HIF-1*α* expression was significantly worse than in those with low expression in patient treated with adjuvant therapy.

Oesophageal cancer is an aggressive tumour with a poor prognosis. Although diagnostic and surgical techniques have been advanced, survival rates have not been improved in the last decade, and the 5-year survival rate of patients with surgically treated oesophageal cancer remains less than 50% in spite of three-field lymph node dissection and combination chemotherapy and radiotherapy (Torres *et al*, 1999; Adham *et al*, 2000; Ando *et al*, 2000; Collard *et al*, 2001).

The choice of therapeutic strategy is based primarily on whether lymph node metastasis has occurred. The presence of lymph node metastasis is the most important determinant of outcome (Ando *et al*, 2000; Kusumi *et al*, 2000). Patients with oesophageal cancer without nodal metastasis have a lower rate of recurrence after operation than those with nodal metastasis (Kato *et al*, 1996; Matsubara *et al*, 1996). However, it is difficult to determine whether lymph node metastases have occurred preoperatively in spite of new imaging techniques. Thus, the identification of a marker that predicts lymph node metastasis, and hence prognosis, is highly desirable.

A hypoxic microenvironment is characteristic of many solid tumours. In the absence of neovascularisation, tumours cannot grow beyond several cubic millimeters, because the diffusion of oxygen, glucose, and other nutrients from blood vessels is limited (Dang and Semenza, 1999). Cancer cell proliferation may outpace the rate of angiogenesis, resulting in tissue hypoxia. To surmount these limitations, the tumour needs to acquire abilities that allow it to adapt to a hypoxic microenvironment (Maxwell *et al*, 1999; Zhong *et al*, 1999).

Hypoxia-inducible-factor 1*α*(HIF-1*α*)is a 120 kDa nuclear protein. Hypoxia-inducible-factor 1 is a heterodimer, consisting of an *α* and a *β* subunit, both belonging to the basic–helix–loop–helix Per-aryl hydrocarbon receptor nuclear translocator-Sim (PAS) family of transcription factors (Wang *et al*, 1995). Hypoxia-inducible-factor-1 is an important component of a widely operative transcriptional response, activated by hypoxia, cobaltous ions, and iron chelation. Hypoxia-inducible-factor 1 activates transcription of hypoxia-inducible genes, including those encoding erythropoietin (Wang and Semenza 1993), vascular endothelial growth factor (VEGF) (Forsythe *et al*, 1996), heme oxygenase-1 (Hoetzel *et al*, 2001), inducible nitric oxide synthase (Jung *et al*, 2000), and the glycolytic enzymes aldolase A, enolase 1, lactate dehydrogenase A (Semenza *et al*, 1996), phosphofructokinase I (Minchenko *et al*, 2002), and phosphoglycerate kinase I (Li *et al*, 1996). The C-terminal of HIF-1*α* binds to p300, and p300/CBP-HIF complexes participate in the induction of hypoxia-responsive genes, including VEGF (Arany *et al*, 1996).

Induction of HIF-1*α* in response to hypoxia is instantaneous, and it can be expressed very early in carcinogenesis, before histologic evidence of angiogenesis or invasion exists (Ryan *et al*, 2000).

It has been shown that HIF-1*α* is a key player in the cancer cells response to low-oxygen tension in a variety of physiologic processes including embryogenesis (Ryan *et al*, 2000), angiogenesis, tumorigenesis (Maxwell *et al*, 1997) and metastases (Zhong *et al*, 1999). Moreover, hypoxic regions have been shown to be both chemo- and radiation resistant (Teicher, 1994; Aebersold *et al*, 2001; Koukourakis *et al*, 2001).

In the current study, we examined archival material from 130 surgical specimens of oesophageal squamous cell carcinoma (OSCC) for HIF-1*α* immunoreactivity. The purpose was to determine the correlation between HIF-1*α* immunoreactivity and clinical and histopathologic factors.

## MATERIALS AND METHODS

### Patients and tissue samples

Surgical specimens were collected from 130 patients with primary OSCC, who underwent radical total oesophagectomy and three-field lymph node dissection from 1989 to 1999 at the Department of Surgical Oncology of Hokkaido University Hospital, Hokkaido Gastroenterology Hospital, or Teine Keijinkai Hospital. Cases of in-hospital death were excluded. The clinicopathologic stage was determined according to the TNM classification system of the International Union Against Cancer (UICC) (Sobin and Wittekind, 1997).

### Immunohistochemistry

The expression of HIF-1*α* was determined immunohistochemically in paraffin-embedded specimens fixed in 10% formalin. Histologic slides, 4 *μ*m in thickness, were deparaffined in xylene and rehydrated through a series of graded ethanol. Endogenous peroxidase activity was blocked by incubation in 3% hydrogen peroxide in methanol for 10 min. The sections were washed twice in phosphate-buffered saline (PBS) and incubated with 10% normal goat serum (Histofine SAB-PO kit, Nichirei Corporation, Tokyo, Japan) for 30 min. The slides were then exposed overnight to a monoclonal antibody against HIF-1*α*(HIF-1*α*Ab-4, NEO MARKERS, Fremont, CA, USA) at a dilution of 1:400 at 4°C. After washing in PBS, a biotinylated goat antibody to mouse immunoglobulin (Histofine SAB-PO kit, Nichirei Corporation) was applied, followed by incubation at room temperature for 60 min. The immunohistochemical reactions were developed in freshly prepared 3,3′-diamino-benzidine tetrahydrochloride (Histofine SAB-PO kit, Nichirei Corporation). Slides were counterstained in haematoxylin and coverslipped in a systemic mounting medium. Tissue samples incubated with nonimmune serum served as negative controls. Immunostaining was evaluated in three visual fields at a power of × 200 under an Olympus microscope (Olympus Optical, Tokyo, Japan).

Tumour cell immunoreactivity to HIF-1*α* protein was scored based on the number of cells exhibiting the nuclear or cytoplasmic staining using the following classification system: −, no staining; 1+, nuclear staining in less than 1% of cells; 2+, nuclear staining in 1%–10% of cells and/or with weak cytoplasmic staining; 3+, nuclear staining in 10% to 50% of cells and/or with distinct cytoplasmic staining; 4+, nuclear staining in more than 50% of cells and/or with strong cytoplasmic staining. Hypoxia-inducible-factor 1*α* 3+and 4+were considered high expression patterns while the remaining cases were considered to be low expression.

All specimens were evaluated by three investigators who were blinded to the patients' clinical information.

### Statistical analysis

Either the *χ*^2^ test or Fisher's exact test was used to analyse the correlation between HIF-1*α* expression and clinicopathologic features. The cumulative survival rate was calculated by the Kaplan–Meier method, and the significance of differences in survival was analysed by the log-rank test. The univariate and multivariate analyses were performed using the Cox proportional hazard regression model; *P*< 0.05 was considered significant in all analyses. Computations were performed using the Statview J version 4.5 (SAS Institute, Inc., Cary, NC, USA) software package.

## RESULTS

### Patient factor

Specimens from 130 patients were included in the current study (113 male and 17 female patients). The median patient age was 63 years (range, 38–82 years). A relatively large number of patients had early-stage disease (81 patients, 62%). Sixty-six patients (51%) had lymph node metastases and 22 patients (17%) had distant nodal metastases. No patient had distant organ metastasis at the time of operation. The study population had the following performance status (PS): PS0, 114 patients; PS1, 15 patients; and PS2, one patient. Following radical operation, adjuvant therapy was administered to 52 patients (Stage I; seven cases, Stage II; 23 cases; Stage III; 11 cases, and Stage IV; 11 cases). Chemotherapy, radiotherapy, and chemoradiotherapy were treated in 12, 18, and 22 patients, respectively (Table 1

**Table 1:** Characteristics of 130 patients with OSCC

**Characteristics**	**Number of patients**
Gender	
Male	113
Female	17
Age	
<60	44
≧60	86
Pathological stage(UICC)	
I	40
IIA	21
IIB	20
III	29
IVA	8
IVB	12
Primary tumour	
T1	56
T2	16
T3	46
T4	12
Regional lymph node	
N0	64
N1	66
Distant Metastasis	
M0	108
M1	22
Histological type	
G1	31
G2	63
G3	36
Performance status	
P0	114
P1	15
P2	1
Adjuvant therapy	
Chemotherapy	12
Radiotherapy	18
Chemoradiotherapy	22
None	78

). The median follow-up period was 29 months (range, 2–114 months).

### Expression of HIF-1*α*

**Figure 1:**
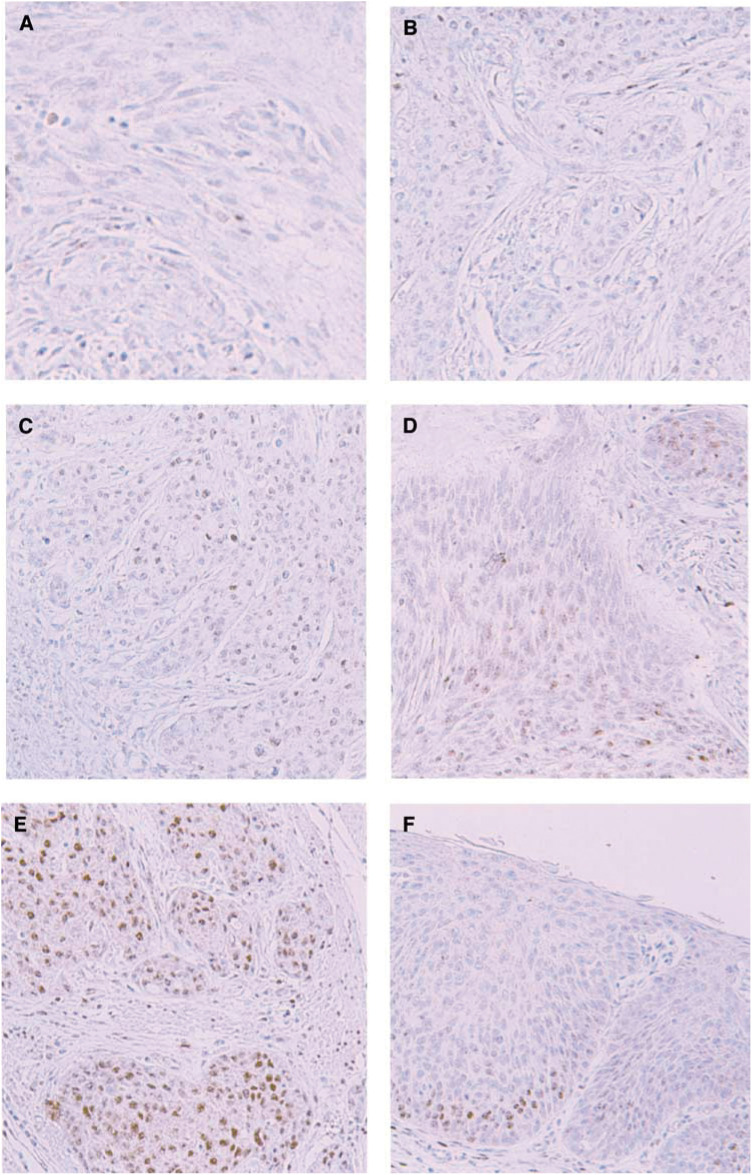
Representative photomicrographs of immunohistochemical staining of HIF1*α* (× 200). Tumour cell immunoreactivity was scored based on nuclear and cytoplasmic staining. (**A**) −, no staining (**B**) 1+, nuclear staining in less than 1% of cells (**C**) nuclear staining in 1-10% of cells and/or with weak cytoplasmic staining (**D**) 3+, nuclear staining in 10-50% of cells and/or with distinct cytoplasmic staining, (**E**) 4+, nuclear staining in more than 50% of cells and/or with strong cytoplasmic staining. (**F**) HIF-1*α*-positive cells are already found in carcinoma *in situ*.

A total of 130 OSCCs were grouped as 42 HIF-1*α* negative tumours; 15 HIF-1*α* 1+tumours; 33 HIF-1*α* 2+tumours; 30 HIF-1*α* 3+tumours; and 10 HIF-1*α* 4+tumours (Figure 1

). Thus, 40 tumours (30.8%) were classified as showing high HIF-1*α* expression.

The frequency of high HIF-1*α* expression increased with tumour stage according to pTNM system: 15.0% of stage I (six of 40 cases), 26.8% of stage II (11 of 41 cases), 44.8% of stage III (13 of 29 cases), and 50.0% of stage IV (10 of 20 cases; Table 2

**Table 2:** Hypoxia-inducible factor 1*α* expression in OSCC by tumour stage

	**HIF-1*α* expression**					
	**Low expression**			**High expression**		
**Histopathologic stage**	**–**	**1+**	**2+**	**3+**	**4+**	**No. of high-expression cases**
Stage I	17	6	11	6	0	6/40(15.0%)
Stage II	17	2	11	9	2	11/41(26.8%)
Stage III	6	3	7	7	6	13/29(44.8%)
Stage IV	2	4	4	8	2	10/20(50.0%)

).

High HIF-1*α* expression correlated with the depth of tumour invasion (*P*=0.0186), lymph node metastasis (*P*=0.0035), distant metastasis (*P*=0.0320), pTNM stage (*P*=0.0019), lymphatic invasion (*P*=0.0492), and positive surgical margin (*P*=0.0156) (Table 3

**Table 3:** Correlation between clinicopathologic features and HIF 1*α* expression in surgical specimens of OSCC

	**HIF-1*α* expression**		
	**Low**	**High**	
**Variable**	**(*n*=40)**	**(*n*=90)**	***P*-value**
Gender			
Male	34	79	0.6646
Female	6	11	
Age			
≧60	22	64	0.0732
<60	18	26	
Double cancer			
Yes	7	19	0.6347
No	33	71	
p-Stage			
I–II	17	64	0.0019
III–IV	23	26	
Histologic grade			
G	11	21	0.6107
Others	29	69	
Depth of tumour invasion			
I–II	16	56	0.0186
III–IV	24	34	
Lymph node metastasis			
N0	12	52	0.0035
N1	28	38	
Distant metastasis			
M0	29	79	0.0320
M1	11	11	
Tumor size (cm)			
<4.5	17	43	0.5774
>4.5	23	47	
Lymphatic invasion			
Positive	27	44	0.0492
Negative	13	46	
Vascular invasion			
Positive	17	26	0.1279
Negative	23	64	
Surgical margin			
Positive	6	3	0.0156
Negative	34	87	
Adjuvant therapy			
Yes	18	34	0.4379
No	22	56	

a TNM classification system of the International Union Against Cancer.

).

HIF-1*α* immunoreactivity had already been identified in carcinoma *in situ* of oesophagus (Figure 1F).

### Kaplan-Meier survival analysis

**Figure 2:**
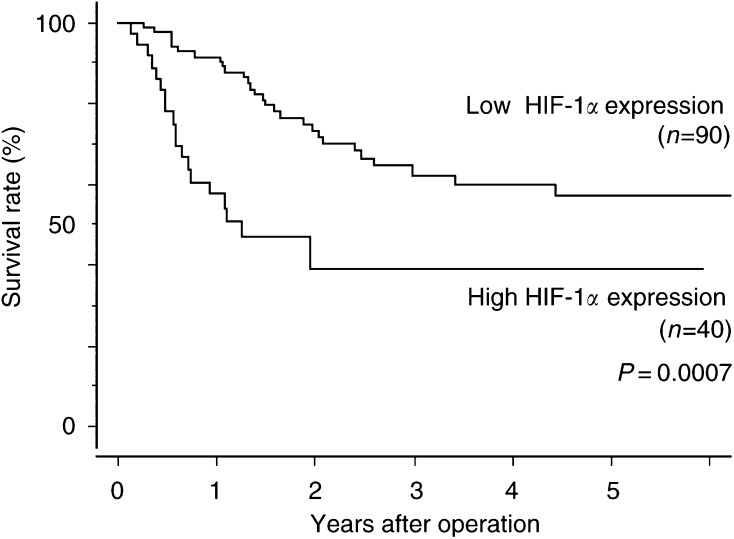
Kaplan–Meier overall survival curves of patients with OSCC with and without high HIF-1*α* expression. *P*=0.0007 by the log-rank test.

The overall 5-year survival rate was 50.4%. The survival curve of patients with a high HIF-1*α* expression tumours was worse than that of patients with low-expression tumours (log-rank test, *P*=0.0007; Figure 2

).

### Univariate survival analysis

Univariate analysis performed by Cox regression identified depth of tumour invasion (*P*<0.0001), distant metastasis (*P*=0.0002), lymph node metastasis (*P*<0.0001), lymphatic invasion (*P*=0.0021), positive surgical margin (*P*<0.0001), and High HIF-1*α* expression (*P*=0.0011) as correlating with survival (Table 4

**Table 4:** Univariate and multivariate analysis of HIF-1*α* and pathologic parameters in patients undergoing curative resection of OSCC

**Factor**	**Hazard ratio (95% CI)**	***P*-value**
*Univaruate*		
HIF-1*α*	2.629 (1.472–4.694)	0.0011
Gender	2.820 (0.877–9.074)	0.0820
Age	1.013 (0.571–1.800)	0.8815
Double cancer	0.700 (0.339–1.445)	0.3347
p-Grade	1.749 (0.786–3.891)	0.1704
Depth of tumour invasion	4.040 (2.215–7.368)	<0.0001
Lymph node metastasis	5.623 (2.843–11.120)	<0.0001
Distant Metastasis	3.269 (1.761–6.068)	0.0002
Tumour size	1.729 (0.965–3.099)	0.0658
Lymphatic invasion	2.565 (1.406–4.681)	0.0021
Vascular invasion	1.536 (0.847–2.784)	0.1577
Surgical margin	5.181 (2.288–11.732)	<0.0001
Adjuvant therapy	1.018 (0.579–1.789)	0.9501

*Multivariate*		
HIF-1*α*	1.539 (0.835–2.837)	0.1669
Depth of tumour invasion	2.646 (1.273–5.499)	0.0091
Lymph node metastasis	4.504 (1.984–10.226)	0.0003
Distant metastasis	1.036 (0.493–2.175)	0.9263
Lymphatic invasion	0.691 (0.314–1.520)	0.3580
Surgical margin	2.634 (1.057–6.561)	0.0375

).

### Multivariate survival analysis

Cox regression multivariate analysis identified depth of tumour invasion (*P*=0.0091), lymph node metastasis (*P*=0.0003), and positive surgical margin (*P*=0.0375), as independent unfavorable factors. High HIF-1*α* expression was not an independent prognostic factor (Table 4).

### Kaplan-Meier survival analysis of the patient treated with adjuvant therapy

**Figure 3:**
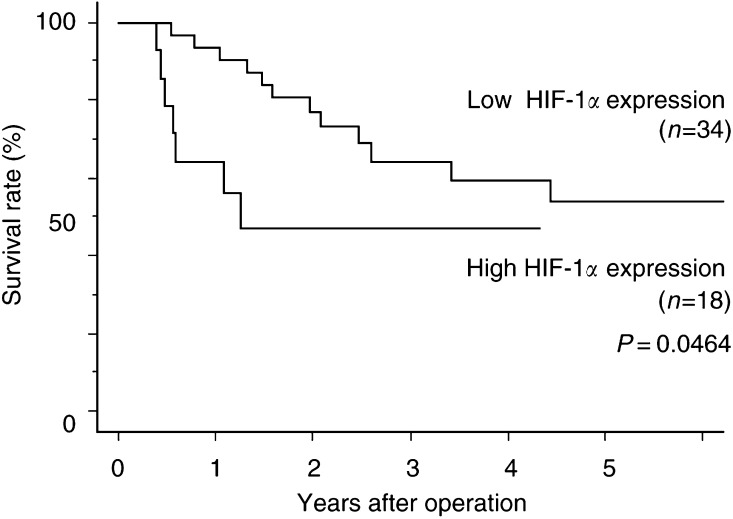
Kaplan–Meier overall survival curves of patients with OSCC underwent adjuvant therapy with or without high HIF-1*α* expression. *P*=0.0464 by the log-rank test.

Survival in patients with a high HIF-1*α* expression pattern was significantly worse than in those with a low-expression pattern in the patient treated with adjuvant therapy (*P*=0.0464; Figure 3

).

## DISCUSSION

The current results show that

1. high HIF-1*α* expression correlates with depth of tumour invasion, lymph node metastasis, distant metastasis, pTNM stage, lymphatic invasion and a positive surgical margin, and
2. survival in patients with a high HIF-1*α* pattern was worse than in those with low-expression pattern.

Although HIF-1*α* was not an independent unfavourable prognostic factor, its expression may strongly influence both tumour proliferation and lymph node metastasis in OSCC. However, it has been reported that HIF-1*α* overexpression was not significantly correlated with pathological parameter in other cancers, including head and neck cancer (Hockel *et al*, 1993; Fyles *et al*, 1998), and oropharyngeal cancer (Aebersold *et al*, 2001). Thus, HIF-1*α* expression seems to behave in a tissue-dependent manner.

Hypoxia has been shown to compromise the beneficial effects of chemotherapeutic drugs (Teicher, 1994) and interfere with the response of tumours to radiation (Moulder and Rockwell, 1987). Pretreatment oxygenation levels have been found to be predictive of the radiation response and survival of patients with cancer of the uterine cervix (Hockel *et al*, 1993; Fyles *et al*, 1998), head and neck (Gatenby *et al*, 1988; Nordsmark *et al*, 1996), oropharyngeal (Aebersold *et al*, 2001), and early oesophageal cancer (Koukourakis *et al*, 2001). In the current study, of all pathological stages, overexpression of HIF-1*α* in OSCC significantly correlates with an unfavourable prognosis in the patients treated with adjuvant therapies.

Preoperative studies on biopsy specimens obtained by endoscopy might allow clinicians to make better-informed therapeutic decisions in conjunction with this marker.

In conclusion, we have suggested that (1) high HIF-1*α* expression may be a marker for lymph node metastasis; and (2) high HIF-1*α* expression may predict an unfavourable prognosis in the patient treated with OSCC.
